# Dataset on migration into EU+2 countries, as well as TB rates and numbers within those countries over the period 2011–2017

**DOI:** 10.1016/j.dib.2019.104042

**Published:** 2019-05-23

**Authors:** D.A. Boudville, R. Joshi, G.T. Rijkers

**Affiliations:** Science Department, University College Roosevelt, Middelburg, the Netherlands

**Keywords:** Tuberculosis, European Union countries, Migrants, Migration

## Abstract

In this data article, TB notification rates in the EU countries along with Iceland and Norway (EU+2 countries) and raw data corresponding to the TB incidence in the period 2011 to 2017 are given. Data on immigration numbers in the EU+2 countries between 2011 and 2017 are also available. Immigration statistics were obtained from a Eurostat Database titled ‘Migration and Migrant Population Statistics’, whereas TB rates were taken from the TB Surveillance and Monitoring Report prepared by European Centre for Disease Control and Prevention in the years 2017, 2018, 2019.

**Table:** Specifications Table

Subject area	*Public Health*
More specific subject area	*Epidemiology*
Type of data	*Table, Excel files and PDF*
How data was acquired	*Eurostat database for Migration and Migrant Population Statistics, European Centre for Disease Prevention and Control: TB Surveillance and Monitoring Reports (2017, 2018, 2019)*
Data format	*Raw*
Experimental factors	*Migration into EU+2 countries 2011-2017* *TB Rate and Numbers in the EU+2 countries 2011-2017*
Experimental features	*Limiting factors to EU+2 countries for period of 2011-2017* *Calculating combined data for EU+2 countries for migration statistics and TB Rate*
Data source location	*University College Roosevelt, Lange Noordstraat 1,* 4331 CB*, Middelburg, Netherlands*
Data accessibility	*Eurostat:**http://appsso.eurostat.ec.europa.eu/nui/show.do?dataset=migr_imm1ctz&lang=en* *European Centre for Disease Prevention and Control:* - *2011:**https://ecdc.europa.eu/sites/portal/files/media/en/publications/Publications/ecdc-tuberculosis-surveillance-monitoring-Europe-2017.pdf* - *2012:**https://ecdc.europa.eu/sites/portal/files/documents/ecdc-tuberculosis-surveillance-monitoring-Europe-2018-rev1.pdf* - *2013–2017:**https://ecdc.europa.eu/sites/portal/files/documents/tuberculosis-surveillance-monitoring-Europe-2019-20_Mar_2019.pdf*
Related research article	*Boudville DA, Joshi R, Rijkers GT. Migration and Tuberculosis in Europe. (Submitted to Tuberculosis)*

**Table:** 

**Value of the data** • Presentation of trends in the immigration numbers and TB incidence in the EU+2 (Iceland and Norway) countries in a tabular form. • Recognition of true contribution of migration to the TB burden in EU+2 countries [1]. • Insight into necessity for future surveillance systems [1].

## 1 Data

This dataset contains tabulated information on migration and tuberculosis in the European Union along with Iceland and Norway (EU+2) in the period 2011 to 2017. The combined data for the EU+2 countries was calculated for both the migration and the TB statistics. All the data was then presented in tabulated form as follows: migration numbers for each EU+2 country, as well as combined, during the period 2011–2017 (Table 1), and TB notification rates for the EU+2 countries and combined along with absolute values for the TB numbers in that same period (Table 2).

**Table 1:** Tuberculosis cases, notification rates per 100,000 total population and mean annual changes in rates for EU+2 (Iceland and Norway), 2011–2017 [3], [4], [5].

Country	2011		2012		2013		2014		2015		2016		2017	
	N	Rate	N	Rate	N	Rate	N	Rate	N	Rate	N	Rate	N	Rate
Austria	684	8.2	646	7.7	653	7.7	586	6.9	583	6.8	634	7.3	570	6.5
Belgium	1019	9.3	976	8.8	963	8.6	949	8.5	977	8.7	1042	9.2	972	8.6
Bulgaria	2406	32.6	2280	31.1	1932	26.5	1872	25.8	1660	23.0	1603	22.4	1463	20.6
Croatia	619	14.4	575	13.4	517	12.1	499	11.7	488	11.5	464	11.1	371	8.9
Cyprus	54	6.4	69	8.0	41	4.7	41	4.8	63	7.4	60	7.1	53	6.2
Czech Republic	600	5.7	597	5.7	497	4.7	511	4.9	517	4.9	516	4.9	505	4.8
Denmark	381	6.9	389	7.0	356	6.4	320	5.7	357	6.3	330	5.8	275	4.8
Estonia	339	25.5	289	21.8	290	22.0	248	18.8	217	16.5	193	14.7	175	13.3
Finland	324	6.0	274	5.1	273	5.0	263	4.8	270	4.9	233	4.2	237	4.3
France	4991	7.7	5003	7.7	4947	7.5	4888	7.4	4744	7.1	4907	7.4	5131	7.7
Germany	4309	5.4	4213	5.2	4340	5.4	4524	5.6	5834	7.2	5949	7.2	5486	6.6
Greece	489	4.4	558	5.0	540	4.9	519	4.7	482	4.4	440	4.1	467	4.3
Hungary	1445	14.5	1223	12.3	1045	10.5	851	8.6	906	9.2	786	8.0	685	7.0
Iceland	9	2.8	11	3.4	11	3.4	9	2.8	7	2.1	6	1.8	14	4.1
Ireland	412	9.0	359	7.8	374	8.1	311	6.7	283	6.1	315	6.7	318	6.6
Italy	4461	7.5	4252	7.2	3973	6.7	3916	6.4	3769	6.2	4032	6.6	3944	6.5
Latvia	885	42.7	993	48.6	904	44.7	761	38.0	721	36.3	660	33.5	552	28.3
Lithuania	1904	62.4	1781	59.3	1705	57.4	1607	54.6	1507	51.6	1441	49.9	1387	48.7
Luxembourg	26	5.1	45	8.6	38	7.1	24	4.4	30	5.3	29	5.0	32	5.4
Malta	33	8.0	42	10.1	50	11.8	46	10.7	32	7.3	50	11.1	42	9.1
The Netherlands	1004	6.0	956	5.7	845	5.0	814	4.8	862	5.1	887	5.2	787	4.6
Norway	354	7.2	374	7.5	392	7.8	323	56.3	313	6.1	295	5.7	261	5.0
Poland	8478	22.3	7542	19.8	7250	19.0	6698	17.6	6430	16.9	6444	17.0	5787	15.2
Portugal	2609	24.7	2606	24.7	2410	23.0	2278	21.8	2196	21.2	1936	18.7	1800	17.5
Romania	19202	95.1	18190	90.5	16689	83.4	15879	79.6	15183	76.4	13601	68.8	13004	66.2
Slovakia	399	7.4	345	6.4	401	7.4	336	6.2	317	5.8	296	5.5	249	4.6
Slovenia	192	9.4	138	6.7	140	6.8	144	7.0	130	6.3	118	5.7	112	5.4
Spain	6798	14.6	6070	13.0	5632	12.1	4913	10.6	5021	10.8	5063	10.9	4570	9.8
Sweden	580	6.2	623	6.6	639	6.7	659	6.8	815	8.4	714	7.2	520	5.2
The United Kingdom	8915	14.1	8711	13.7	7870	12.3	7029	10.9	6224	9.6	6116	9.4	5567	8.5
Combined	73921	14.5	70130	13.8	65717	12.8	61818	12.1	60938	11.9	59161	11.5	55336	10.7

**Table 2:** Total number of long-term immigrants arriving into the reporting country during the reference year [2].

Country	2011	2012	2013	2014	2015	2016	2017
Austria	82230	91557	101866	116262	166323	129509	111801
Belgium	147377	129477	120078	123158	146626	123702	126703
Bulgaria	:	14103	18570	26615	25223	21241	25597
Croatia	8534	8959	10378	10638	11706	13985	15553
Cyprus	23037	17476	13149	9212	15183	17391	21306
Czech Republic	27114	34337	30124	29897	29602	64083	51847
Denmark	52833	54409	60312	68388	78492	74383	68579
Estonia	3709	2639	4109	3904	15413	14822	17616
Finland	29481	31278	31941	31507	28746	34905	31797
France	319816	327431	338752	340383	364221	378115	369964
Germany	489422	592175	692713	884893	1543848	1029852	917109
Greece	60089	58200	57946	59013	64446	116867	112247
Hungary	28018	33702	38968	54581	58344	53618	68070
Iceland	4073	4960	6406	5368	5635	8710	12116
Ireland	57292	61324	65539	73519	80792	85185	78499
Italy	385793	350772	307454	277631	280078	300823	343440
Latvia	10234	13303	8299	10365	9479	8345	9916
Lithuania	15685	19843	22011	24294	22130	20162	20368
Luxembourg	20268	20478	21098	22332	23803	22888	24379
Malta	5465	8256	10897	14454	16936	17051	21676
The Netherlands	130118	124566	129428	145323	166872	189232	189646
Norway	70337	69908	68313	66903	60816	61460	53351
Poland	157059	217546	220311	222275	218147	208302	209353
Portugal	19667	14606	17554	19516	29896	29925	36639
Romania	147685	167266	153646	136035	132795	137455	177435
Slovakia	4829	5419	5149	5357	6997	7686	7188
Slovenia	14083	15022	13871	13846	15420	16623	18808
Spain	371331	304053	280772	305454	342114	414746	532132
Sweden	96467	103059	115845	126966	134240	163005	144489
The United Kingdom	566044	498040	526046	631991	631452	588993	644209
Combined	3348090	3394164	3491545	3860080	4725775	4353064	4461833

## 2 Experimental design, materials, and methods

### 2.1 Migration numbers

Statistics for number of long-term immigrants into the EU+2 countries was obtained from’ Migration and Migrant Population Statistics’ database from Eurostat [2]. The dataset was filtered to include the immigration numbers for EU+2 countries between the years 2011–2017. The total number of immigrants per year for the EU+2 countries was calculated by summing up numbers for each country in the respective year.

### 2.2 TB rate

To obtain data for TB notification rates per 100,000 population for the 30 countries, the Surveillance and Monitoring reports from the year 2017, 2018 and 2019 were used to obtain the most up-to-date data [3], [4], [5]. The table from the ECDC report was filtered to exclude data for all other countries in Europe except EU countries and Iceland and Norway. TB rates for year 2011 was obtained from Surveillance report from 2017 [3]. TB rate for year 2012 was taken from Surveillance report published in the 2018 [4], whereas TB rates for years 2013–2017 was obtained from Surveillance report from the year 2019 [5]. For the overall TB rates for the 30 countries, number of TB cases per year were summed up and rates were calculated per 100,000 population in the EU+2 countries.
