# Supplementary material for: Dataset on migration into EU+2 countries, as well as TB rates and numbers within those countries over the period 2011–2017
Source: Data Brief. 2019 May 23;25:104042. doi: 10.1016/j.dib.2019.104042 (PMC6582061; doi:10.1016/j.dib.2019.104042)
Supplement: Supplementary file 1 — Multimedia component 1 [file mmc1.docx]

Conflict of Interest and Authorship Conformation Form

Please check the following as appropriate:

- All authors have participated in (a) conception and design, or analysis and interpretation of the data; (b) drafting the article or revising it critically for important intellectual content; and (c) approval of the final version.
- This manuscript has not been submitted to, nor is under review at, another journal or other publishing venue.
- The authors have no affiliation with any organization with a direct or indirect financial interest in the subject matter discussed in the manuscript
- The following authors have affiliations with organizations with direct or indirect financial interest in the subject matter discussed in the manuscript:

Author’s name Affiliation

Danielle A. Boudville *Science Department, University*

*College Roosevelt, Lange Noordstraat 1, 4331 CB, Middelburg, Netherlands*

Richa Joshi *Science Department, University*

*College Roosevelt, Lange Noordstraat 1, 4331 CB, Middelburg, Netherlands*

Ger Rijkers *Science Department, University College Roosevelt, Lange Noordstraat 1, 4331 CB, Middelburg, Netherlands*

*Laboratory for Medical Microbiology and Immunology, St Elisabeth Hospital, Tilburg, Netherlands*
